# Influence of human population movements on urban climate of Beijing during the Chinese New Year holiday

**DOI:** 10.1038/srep45813

**Published:** 2017-03-30

**Authors:** Jingyong Zhang, Lingyun Wu

**Affiliations:** 1 Center for Monsoon System Research, Institute of Atmospheric Physics, Chinese Academy of Sciences, Beijing 100029, China; 2 University of Chinese Academy of Sciences, Beijing, 100049, China; 3 State Key Laboratory of Numerical Modeling for Atmospheric Sciences and Geophysical Fluid Dynamics (LASG), Institute of Atmospheric Physics, Chinese Academy of Sciences, Beijing 100029, China

## Abstract

The population movements for the Chinese New Year (CNY) celebrations, known as the world’s largest yearly migration of human beings, have grown rapidly in the past several decades. The massive population outflows from urban areas largely reduce anthropogenic heat release and modify some other processes, and may thus have noticeable impacts on urban climate of large cities in China. Here, we use Beijing as an example to present observational evidence for such impacts over the period of 1990–2014. Our results show a significant cooling trend of up to 0.55 °C per decade, particularly at the nighttime during the CNY holiday relative to the background period. The average nighttime cooling effect during 2005–2014 reaches 0.94 °C relative to the 1990s, significant at the 99% confidence level. The further analysis supports that the cooling during the CNY holiday is attributable primarily to the population outflow of Beijing. These findings illustrate the importance of population movements in influencing urban climate despite certain limitations. As the world is becoming more mobile and increasingly urban, more efforts are called for to understand the role of human mobility at various spatial and temporal scales.

Cities house around 54% of the world’s population, and play a central role in the global sustainable development^1^. Cities, accounting for more than 70% of carbon emission, are the main driver of global climate change^2^. Large human populations, extensive impervious surfaces and congested traffic conditions alter local urban climate in many ways^3^,^4^. Urban heat island (UHI), referring to an urban area with higher surface air temperature than its surroundings, occurs in almost all cities across the globe^5^,^6^,^7^,^8^,^9^. The UHI effects are mainly caused by the man-made surface and anthropogenic heating^3^,^10^. The anthropogenic heat release is of particular importance at mid- and high-latitude cities during the wintertime due to smaller solar radiation and greater energy consumption^11^,^12^,^13^. UHI overlapped with CO_2_-induced global warming influences not only urban climate, but also environmental quality, biodiversity, and human well-beings^14^,^15^,^16^,^17^,^18^.

The people become increasingly mobile at various spatial and temporal scales. Between 1950 and 2014, more than 3.1 billon people migrated from rural to urban communities^1^. As the planet continues to urbanize, another 2.5 billion people are expected to be added to the urban population by 2050. The international tourist arrivals have grown rapidly since 1950, from 25 million to 1184 million in 2015, and are projected to reach 1.8 billion by 2030^19^. However, how or to what extent human mobility can affect urban climate remained generally overlooked.

The mass human migration during the Chinese New Year (CNY) or Spring Festival holiday, which is mainly triggered by migrating workers with their permanent residence in rural communities^20^, represents the largest periodic migration of human beings across the globe. Hundreds of millions of Chinese people living in large cities go back their home towns and villages or travel for pleasure to celebrate the most important festival in China. It was estimated that during the CNY holiday season, passenger journeys nationwide have increased from about 1 billion in the early 1990s to 3 billion or more in recent years. The massive population outflows from large cities during the CNY holiday can largely reduce anthropogenic heat release and also alter some other processes, and may thus exert noticeable impacts on urban climate. Indeed, recent studies demonstrated that the massive population outflows can have the cooling effects on urban temperature^21^,^22^,^23^. It was evident that UHI effect of Beijing averaged over 2009–2013 declined by 0.64 °C during the CNY holiday week relative to the nearby non-holiday period as the anthropogenic heat release decreased substantially^21^. However, this research is based on the analysis to two urban stations and two non-urban reference stations for a short period. More robust evidence is clearly needed. Here, we utilize a new method to assess the impacts of mass human migration on urban surface air temperature of Beijing during the CNY holiday for the period of 1990–2014.

We estimate population movement effects on urban surface air temperature of Beijing based on analysis of data from available four urban stations and two non-urban reference stations for 1990–2014 (see Data and Methods for detail, Fig. 1). We firstly calculate the UHI intensity as surface air temperature difference between urban and non-urban stations to minimize the effects of regional atmospheric circulation conditions and isolate the local urban effects^24^. For each day of each year, we further obtain the anomaly of the UHI intensity by subtracting 1990–2014 climatic mean UHI intensity on the same date from the original value to reduce the effect of the seasonal variability. Then, we examine the trend in the differences of UHI anomalies between the CNY holiday and the background period (Fig. S1) to identify the impacts of population movements on urban daily mean surface air temperature (T_mean_), daily maximum surface air temperature (T_max_) and daily minimum surface air temperature (T_min_). Since the CNY holiday and the background period share the same urban surface properties (buildings, roads, and so on) every year, the differences of UHI anomalies between the two periods help to minimize the effects of UHI changes associated with the increasing urban surface areas from 1990 to 2014. As the population outflows have grown rapidly, we choose the first and last 10 years (1990s and 2005–2014) of data to represent the cases with the least and the most likely impacts, respectively, and further compare the difference between the two periods. Note that the resulting changes in urban T_mean_, T_max_ and T_min_ are relative to the non-urban values. Finally, we examine the dependence of urban T_mean_, T_max_, and T_min_ variations induced by the differences of UHI anomalies between the CNY holiday and the background period on the floating population and anthropogenic heating reduction.

## Results

### Cooling effects of population movements during the CNY holiday

Beijing, as the capital of China and one of the world’s largest cities, had undergone rapid urbanization during 1990–2014. The floating population of Beijing had grown at an unprecedented rate, from 0.54 million in 1990 to 8.19 million in 2014 (Fig. 2a). The floating people do not have permanent residence in Beijing, and usually choose to return to their home towns or villages before the CNY holiday^25^. This is particularly true in recent years given the fast economic development and improved transportation. The outflow of people from Beijing was estimated to reach around 9 million during the CNY holiday of 2014. During the background period, the daily mean, daytime and nighttime UHI anomalies with respect to the 1990–2014 climatic means in Beijing exhibit warming trends of 0.09–0.49 °C per decade (Fig. S2). In contrast, the daily mean, daytime and nighttime UHI anomalies during the CNY holiday all have cooling trends. The trend in the differences of UHI anomalies between the CNY holiday and the background period is further calculated to estimate population outflow impacts on urban surface air temperature of Beijing during the CNY holiday. As the population outflow of Beijing increased rapidly, we identify a significant cooling trend of 0.36 °C per decade (p < 0.05) with respect to urban T_mean_ during the CNY holiday for the period of 1990–2014 (Fig. 2b). Urban T_min_ shows a much larger decreasing trend of 0.55 °C per decade (p < 0.01) than that of urban T_max_ with a magnitude of 0.22 °C per decade (p < 0.05) (Fig. 2c,d). This indicates that the cooling during the CNY holiday is much stronger at the nighttime than at the daytime.

### Difference in population movement impacts between 2005–2014 and the 1990s

China’s urbanization policies mainly focused on developing small and medium cities during the 1990s, and then emphasized the coordinated development of cities across various scales^26^. As a result, the urban growth rate of Beijing was relatively slow during the 1990s, and has speeded up since 2000. Averaged over the 1990s, only 1.12 million floating people lived in Beijing, merely occupying 9.5% of the total population (Fig. 2a). Therefore, the effects of population movements on urban climate should be small during this period. In contrast to the 1990s, the floating population during 2005–2014 increased sharply, reaching 6.22 million and 33% in absolute and relative terms. The effects of population movements are, therefore, expected to be much stronger during 2005–2014 than during the 1990s. We further compare the difference in population movement impacts between 2005–2014 and the 1990s (Fig. 3). Relative to the 1990s, the 2005–2014 average urban T_mean_ decreases by 0.64 °C. The asymmetric cooling effects at the daytime and nighttime also show up, with the decreasing magnitudes of 0.34 °C and 0.94 °C for urban T_max_ and urban T_min_, respectively, during 2005–2014 relative to the 1990s.

### Strong dependence of the cooling during the CNY holiday on the floating population and anthropogenic heating reduction

Population movements around the CNY are determined by, and increase consistently with the floating people^25^. Due to the lack of long-term statistical data for how many people leave Beijing during the CNY holiday, we take the floating population as an indicator of the population outflow. Large year-to-year variations seen in urban T_mean_, T_max_, and T_min_ induced by the differences of UHI anomalies between the CNY holiday and the background period (Fig. 2b–d) may be unrelated with the impacts of the continuously increasing population movements in general. We further examine the dependence of the cooling during the CNY holiday on the floating population and anthropogenic heating index after a 9-year moving average is applied to reduce the signal of interannual variability such as that resulting from surface sea temperature anomalies and meteorological conditions^3^,^8^,^24^ (see Data and Methods for detail).

The tight coupling is seen between increasing floating population and decreasing urban T_mean_ with the R^2^ value of the linear fit reaching up to 84%, pointing to that the population outflow is primarily responsible for the cooling during the CNY holiday (Fig. 4a). The coupling is stronger for urban T_min_ than for urban T_max_ with the R^2^ values of 90% and 63%, respectively, corresponding well with the stronger cooling at the nighttime than at the daytime (Fig. 4c,e). Figure 4b shows that the urban T_mean_ decrease during the CNY holiday depends strongly on anthropogenic heating reduction with the R^2^ value of 85%, meaning that anthropogenic heat release reduction plays a vital role for the cooling during the CNY holiday. Again, the dependence is stronger at the nighttime than at the daytime (Fig. 4d,f). We also perform the same analysis as Fig. 4 except that 6-to-8-year moving averages are applied. The R^2^ values for urban T_mean_ decrease when smaller moving average intervals are applied, but are still all higher than 66% (Table S2). The results consistently suggest that the cooling during the CNY holiday is mainly controlled by the population outflow. Also, urban T_min_ is more closely tied to the floating population and anthropogenic heating reduction than urban T_max_. The above results support that the cooling of urban surface air temperature during the CNY holiday can be attributed primarily to the impacts of population movements. Meanwhile, the limitations that only annual population and energy consumption data are available and used in this study should be recognized, and more detailed analyses are needed to be addressed in future when more statistical data become available.

## Summary and Discussion

To conclude, the population outflow during the CNY holiday which can cause less anthropogenic heat release and also affect some other processes, has the cooling effects on urban surface air temperature of Beijing, with a much stronger magnitude at the nighttime than at the daytime. The further analysis provides confidence that the cooling during the CNY holiday is due primarily to the population outflow of Beijing. Meanwhile, this study should be considered as illustrative rather than definitive as it has certain limitations. With large population influx, the whole plain area of Beijing and its adjacent area have more or less urbanized. The two reference stations actually represented the mixed urban and rural surroundings of metropolitan Beijing, and also experienced some population outflow during the CNY holiday. As a result, the cooling effects of massive population movements might be underestimated. And as some people choose to leave Beijing before the Spring Festival travel season and return after that, urban surface air temperature in the background period was also influenced by the population outflow to some degree. This may further exaggerate the underestimation. Furthermore, some firework and firecrackers usually occur during the CNY holiday, which may bias our estimates though the effects are probably small regarding the half-month average. In addition, it is noted that urban T_mean_, T_max_ and T_min_ all show warming effects in the Olympic year of 2008. Some environmental control measurements implemented for the Olympic Games early since the beginning of this year might have contributed to this phenomenon. When excluding the Olympic year, the 2005–2014 average cooling effects increased by 0.04–0.18 °C (Fig. 3).

Our findings present observational evidence for the short-term cooling impacts of population movements as anthropogenic heat release decreased substantially. Moreover, population movements are implicated in regional and global climate systems in a longer-term manner^2^,^27^. Rural-to-urban migration is a key player in driving land use changes, transforming rural landscapes and extending urban areas^28^,^29^. These changes can subsequently rise surface air temperature, change precipitation pattern, and modulate other aspects of regional climate system by altering biophysical and biochemical processes^30^. The increased green-house gas emissions directly and indirectly resulting from population movements contribute considerably to global warming^31^. Also, larger human mobility can produce more air and water pollutions, noise, and artificial light, and create many environmental challenges.

As cities continue to expand in size and number, urban climate will increasingly influence the human society. Our estimates represent population movement effects on a certain aspect of urban climate in a short-term manner. However, given the increased human mobility globally, this study raises concerns about an important yet long neglected issue that calls for further investigation. We need to better understand this system and its dynamics at multiple spatial and temporal scales, and future urban planning, design and management should account for the role of human mobility to improve urban sustainability and livability and promote the well-beings of city dwellers.

## Data and Methods

### Data

**Figure 1:**
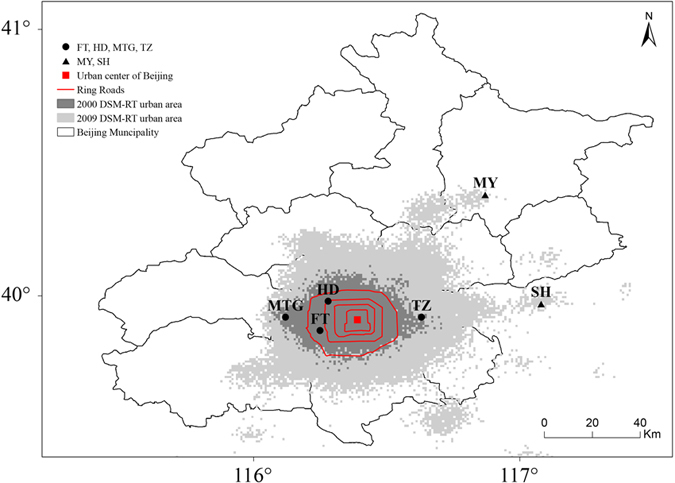
Locations of 4 urban stations and 2 non-urban reference stations. Specifically, urban stations include Fengtai (FT), Haidian (HD), Mentougou (MTG), Tongzhou (TZ), and 2 non-urban reference stations include Miyun (MY), and Sanhe (SH). The red rectangle indicates the urban center of Beijing. The black lines denote the districts of Beijing, and the 2nd-5th Ring Roads in Beijing are marked by red lines. Dark grey and light grey areas represent urban extents of Beijing in 2000 and 2009, respectively. Map was generated using ArcGIS10.3 (www.esri.com/software/arcgis).

**Figure 2:**
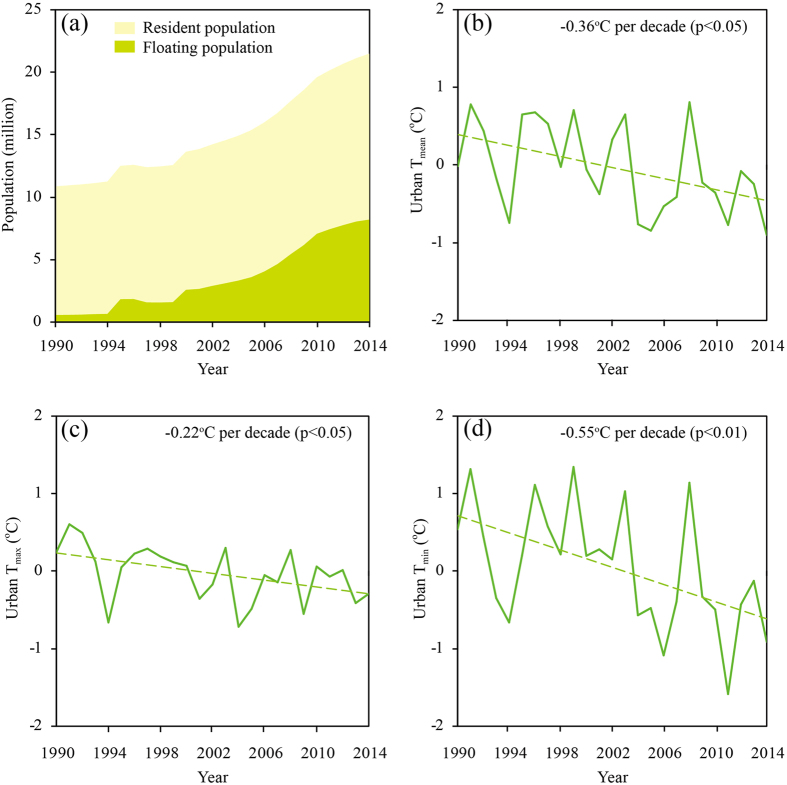
(**a**) Interannual variations of Beijing’s total resident population and floating population for the period of 1990–2014. Interannual variations of (**b**) urban daily mean surface air temperature (T_mean_), (**c**) urban daily maximum surface air temperature (T_max_), and (**d**) urban daily minimum surface air temperature (T_min_) induced by the differences in urban heat island (UHI) anomalies between the Chinese New Year (CNY) holiday and the background period (CNY holiday minus the background period). The UHI is calculated as surface air temperature difference between 4 urban stations and 2 non-urban reference stations. Note that the UHI anomaly represents a change relative to the 1990–2014 climatic mean. The urban T_mean_, T_max_, and T_min_ variations are relative to the non-urban values. The trend (dashed line) is used to assess population movement impact on urban surface air temperature.

**Figure 3:**
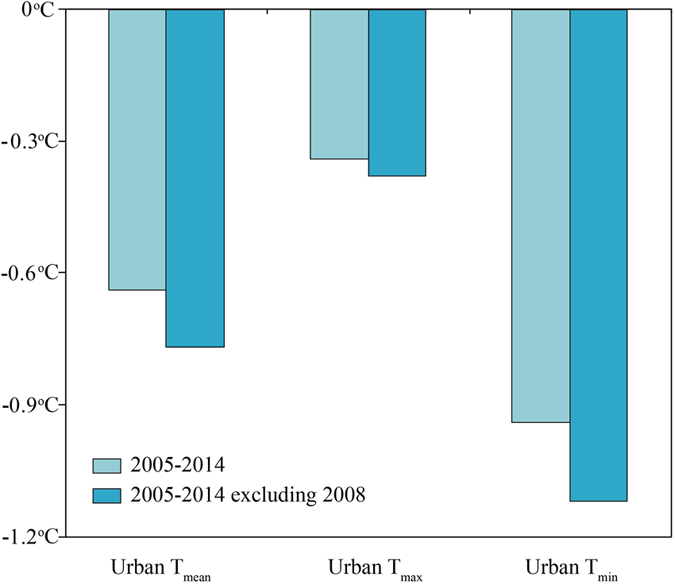
The population movement impacts on urban daily mean surface air temperature (T_mean_), daily maximum surface air temperature (T_max_), and daily minimum surface air temperature (T_min_) during 2005–2014 relative to the 1990s. The light and dark blue bars represent the values with and without the Olympic year of 2008 included, respectively. The difference of urban heat island (UHI) anomalies between the Chinese New Year (CNY) holiday and the background period averaged over 2005–2014 minus that averaged over the 1990s is used to assess the population movement impact on urban surface air temperature. The urban T_mean_, T_max_, and T_min_ changes are relative to the non-urban values.

**Figure 4:**
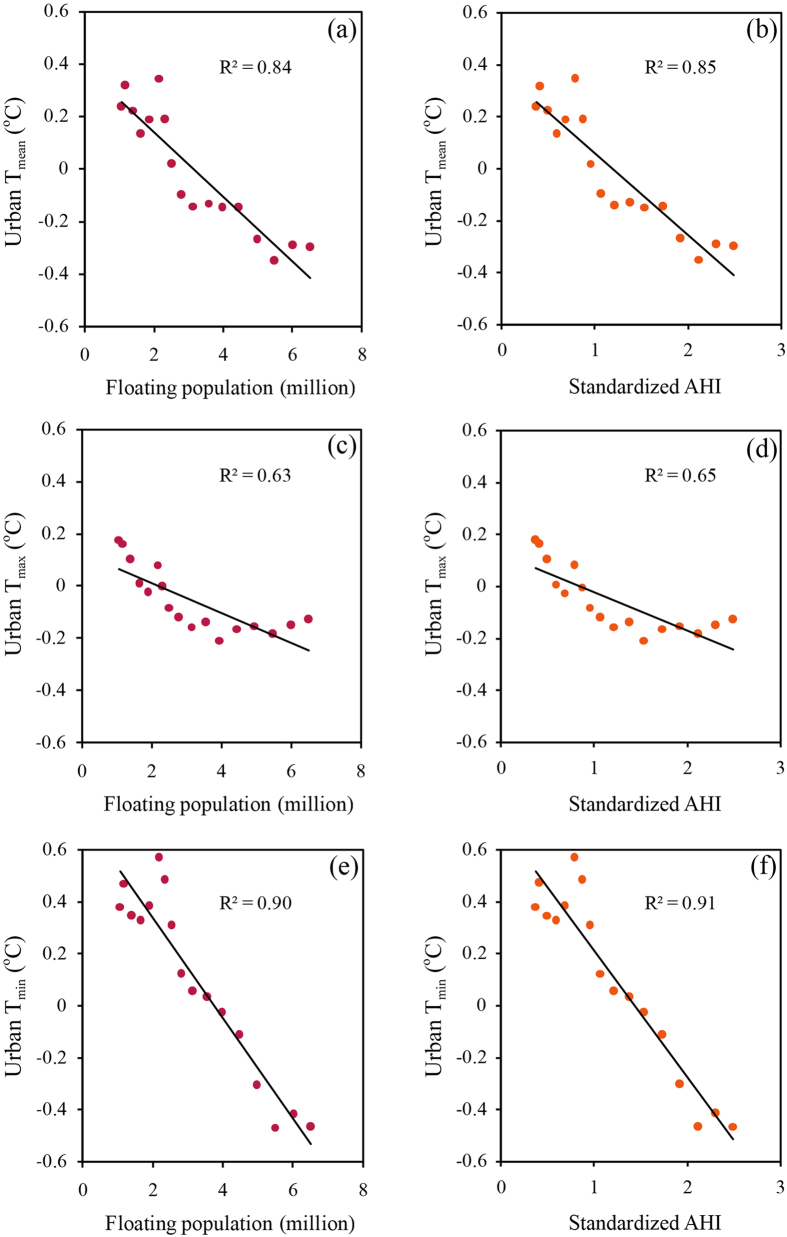
Dependence on the floating population (left panel) and anthropogenic heating index (AHI, right panel) of (**a,b**) urban daily mean surface air temperature (T_mean_), (**c,d**) urban daily maximum surface air temperature (T_max_), and (**e,f**) urban daily minimum surface air temperature (T_min_) induced by the differences in urban heat island (UHI) anomalies between the Chinese New Year (CNY) holiday and the background period. The 9-year moving average is applied to all data. The solid line represents the linear fit, and the goodness of fit (R^2^) is also shown.

We take daily mean, maximum, and minimum surface air temperature data at 2474 weather stations across China from the China Meteorological Administration. Among them, 20 stations are located in Beijing. We only consider the stations without any relocation over the study period of 1990–2014, and further remove the stations with elevations higher than 100 m to reduce the effect of the topography^32^. As a result, four stations located in populous urban areas are used in this study (Fig. 1). Specifically, they are Haidian (HD), Fengtai (FT), Mentougou (MTG), and Tongzhou (TZ) stations. We use Miyun (MY) station of Beijing and Sanhe (SH) station of Hebei province as non-urban reference stations, both located in the plain areas with low population density. The data of resident and floating populations and energy consumption are obtained from the Beijing Municipal Bureau of Statistics (http://www.bjstats.gov.cn). The floating people live in Beijing, but have permanent residence in rural communities and are not included in the Beijing household registration system. The data of urban areas in 2000 and 2009 as determined from QuikSCAT/DSM-RT data are provided by Jacobson *et al*.^33^.

### The CNY holiday and the background period

The CNY or Spring Festival refers to the first day of the lunar-calendar month, therefore, its solar-calendar date varies each year. During the study period of 1990–2014, it falls on the new moon between 22 January and 19 February (Table S1). The CNY celebrations traditionally persist from the evening preceding the CNY day to the 15th day of the first lunar-calendar month known as the Lantern Festival or YuanXiao Festival. We therefore define the CNY holiday as from the CNY day to the 15th day of the first lunar-calendar month (day +1 to day +15). The Spring Festival travel season or Chunyun usually lasts for 40 days, from 15 days before to 24 days after the CNY day. We exclude this period, and define the background period as from 45 days to 16 days before the CNY day (day −45 to day −16), and from 25 days to 54 days after (day +26 to day +55) (Fig. S1). In total, there are 60 days.

### Statistical analysis

As the urban areas and their surroundings usually share the same regional atmospheric circulation and weather conditions, the UHI primarily reflects the local urban effects^24^. We firstly calculate the UHI intensity of Beijing by comparing surface air temperature difference between four urban stations and two non-urban stations to reduce the effects of the regional atmospheric circulation conditions. Because the UHI effects of Beijing have obvious seasonal change, we remove the 1990–2014 mean daily values from the original daily UHI series for each year. We thus obtain daily UHI anomalies with respect to the 25-year mean seasonal cycle. As an example, Figure S3 presents the UHI values from 17 December to 26 March (day −45 to day +55) in 2014, the UHI values on the same dates as in 2014 but averaged over the period of 1990–2014, and their differences (the UHI anomalies in 2014). As the floating people and the population outflows during the CNY holiday increased rapidly, we calculate the linear trend of the differences in UHI anomalies between the CNY holiday and the background period to estimate the population movement impacts on urban surface air temperature of Beijing. Note that the UHI differences between the CNY holiday and the background period implicitly minimize the effects of the UHI seasonality even though the removal of 1990–2014 mean daily UHI values is unapplied. We compare linear trends and annual values of the UHI differences between the CNY holiday and the background period with and without the removal of the 1990–2014 mean daily UHI values, and find that linear trends only have very small differences while annual values differ to some degree (Fig. S4). The population movement impacts are expected to be largest in the last several years, and we further compare the difference between the last 10 years (2005–2014) and the first 10 years (1990s) of the study period. Given the possible effects of some environmental control measurement implemented in the Olympic year of 2008, we also examine the difference with and without this year excluded.

We further examine the dependence of urban T_mean_, T_max_, and T_min_ variations induced by the differences in UHI anomalies between the CNY holiday and the background period on the floating population that is used as an indicator of the population outflow during the CNY holiday, and the anthropogenic heating reduction. Since only annual energy consumption and population data are available (http://www.bjstats.gov.cn), we calculate the annual anthropogenic heating (Q) as the sum of heat produced by the total energy consumption (Q_E_) and heat release directly from human metabolism (Q_H_)^34^.


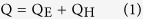


Q_E_ and Q_H_ are calculated as follows:


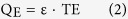


where ε = 2.9306 × 10^10^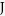 is the heat emission per ton of standard coal equivalent (tce)^35^,^36^, and TE represents the total energy consumption in unit of tce.


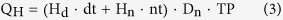


where H_d_ and H_n_ denote the human metabolic rates during the daytime (dt, 0600–2200: 57600 s) and the nighttime (nt, 2200–0600: 28800 s), respectively. We use typical values of 180 J/s for H_d_ and 70 J/s for H_n_^37^. D_n_ is the total days of a year and TP is the total resident population.

We further develop an anthropogenic heating index (AHI) to roughly reflect anthropogenic heating reduction during the CNY holiday.


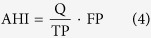


where FP represents the floating population. The AHI is standardized by one standard deviation. The dependence of urban T_mean_, T_max_, and T_min_ variations induced by the differences of UHI anomalies between the CNY holiday and the background period on the floating population and standardized AHI are examined after a 9-year moving average is applied to reduce the signal of interannual variability which may be unrelated with the population movement impacts. We also conducted the same analysis but with 6-to-8-year moving averages applied. All statistical analyses were performed in S-plus version 8.1.1.

## Additional Information

**How to cite this article**: Zhang, J. and Wu, L. Influence of human population movements on urban climate of Beijing during the Chinese New Year holiday. *Sci. Rep.* **7**, 45813; doi: 10.1038/srep45813 (2017).

**Publisher's note:** Springer Nature remains neutral with regard to jurisdictional claims in published maps and institutional affiliations.

## Supplementary Material

Supporting Tables and Figures
