# Supplementary material for: Influence of human population movements on urban climate of Beijing during the Chinese New Year holiday
Source: Sci Rep. 2017 Mar 30;7:45813. doi: 10.1038/srep45813 (PMC5372473; doi:10.1038/srep45813)
Supplement: Supporting Tables and Figures [file srep45813-s1.doc]

**Supporting Tables and Figures**

**Influence of human population movements on urban climate of Beijing during the Chinese New Year holiday**

Jingyong Zhang^1,2^ and Lingyun Wu^3^

1 Center for Monsoon System Research, Institute of Atmospheric Physics, Chinese Academy of Sciences, Beijing 100029, China

2 University of Chinese Academy of Sciences, Beijing, 100049, China

3 State Key Laboratory of Numerical Modeling for Atmospheric Sciences and Geophysical Fluid Dynamics (LASG), Institute of Atmospheric Physics, Chinese Academy of Sciences, Beijing 100029, China

**List of supporting tables and figures**

Supporting Table S1

Supporting Table S2

Supporting Figure S1

Supporting Figure S2

Supporting Figure S3

Supporting Figure S4

**Table S1.**  Date of Chinese New Year (CNY) during the period of 1990-2014

| Date | Year |
| --- | --- |
| 22 January | 2004 |
| 23 January | 1993, 2012 |
| 24 January | 2001 |
| 26 January | 2009 |
| 27January | 1990 |
| 28 January | 1998 |
| 29 January | 2006 |
| 31 January | 1995, 2014 |
| 1 February | 2003 |
| 3 February | 2011 |
| 4 February | 1992 |
| 5 February | 2000 |
| 7 February | 1997, 2008 |
| 9 February | 2005 |
| 10 February | 1994, 2013 |
| 12 February | 2002 |
| 14 February | 2010 |
| 15 February | 1991 |
| 16 February | 1999 |
| 18 February | 2007 |
| 19 February | 1996 |

**Table S2.** The R^2^ values for the linear fit of urban daily mean surface air temperature (T_mean_ ), daily maximum surface air temperature (T_max_), and daily minimum surface air temperature (T_min_) variations induced by the differences of urban heat island (UHI) anomalies between the Chinese New Year (CNY) holiday and the background period against the floating population and standardized anthropogenic heat index (AHI) with 6-to-9-year moving averages applied. The R^2^ denotes the goodness of fit.

| Urban temperature | Floating population and AHI | 9-year | 8-year | 7-year | 6-year |
| --- | --- | --- | --- | --- | --- |
| T_mean_ | Floating population | 0.84 | 0.77 | 0.70 | 0.66 |
|  | standardized AHI | 0.85 | 0.79 | 0.73 | 0.68 |
| T_max_ | Floating population | 0.63 | 0.55 | 0.48 | 0.44 |
|  | standardized AHI | 0.65 | 0.57 | 0.50 | 0.46 |
| T_min_ | Floating population | 0.90 | 0.85 | 0.79 | 0.74 |
|  | standardized AHI | 0.91 | 0.86 | 0.81 | 0.75 |


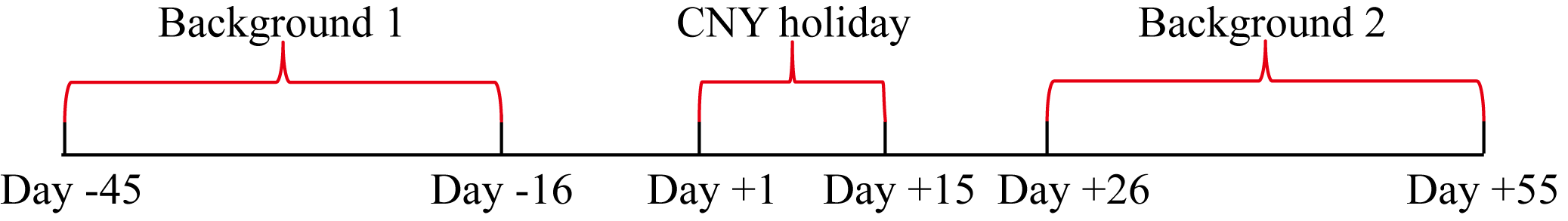


**Figure S1.** Definition of the Chinese New Year (CNY) holiday and the background period. The CNY holiday refers to from the CNY day to the 15th day of the first lunar-calendar month (Day +1 to Day +15). The background period is defined as 60 days including 45 days to 16 days before the CNY day (Day -45 to Day -16) and 25 days to 54 days after (Day +26 to Day +55).


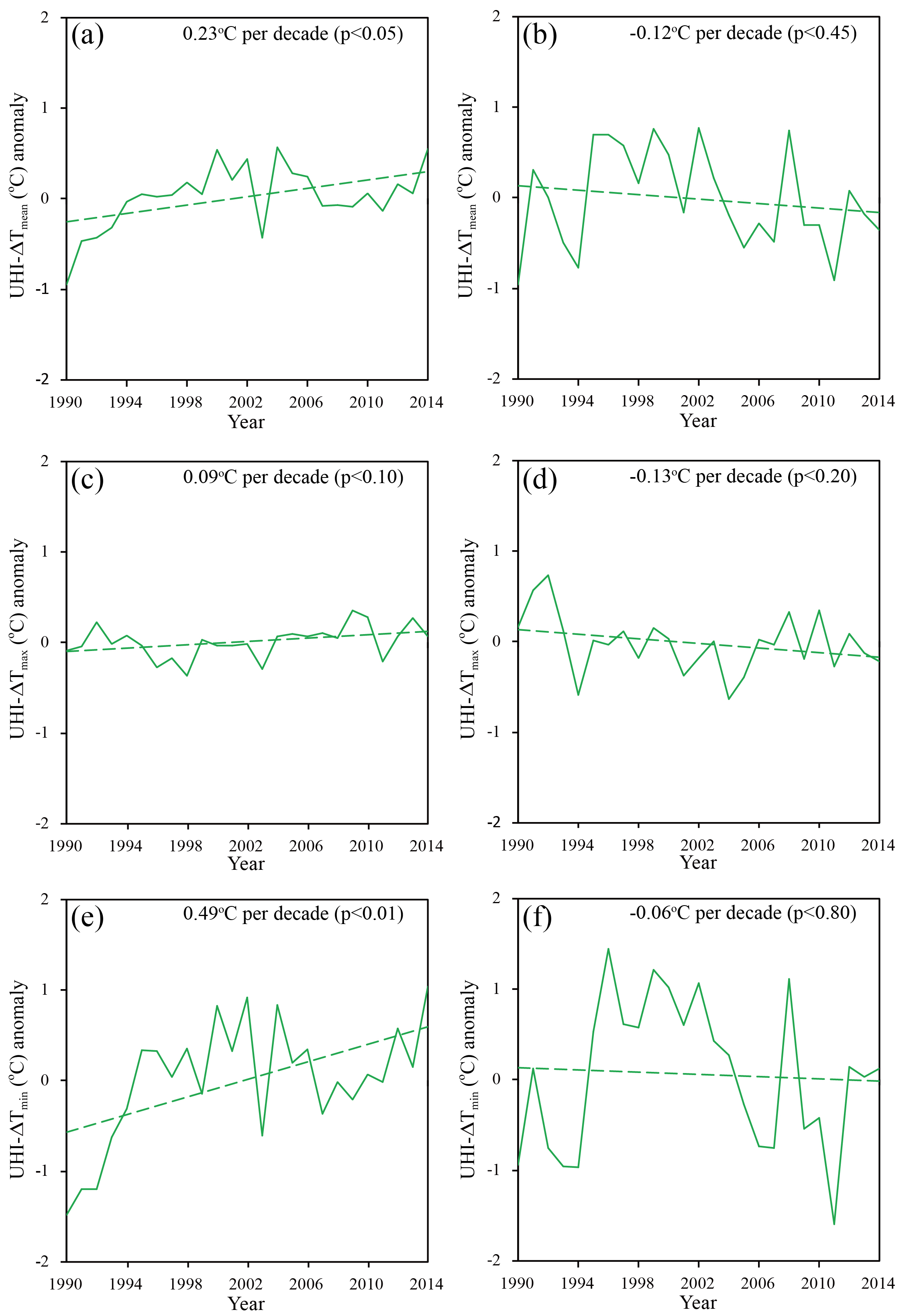


**Figure S2.** Interannual variations of urban heat island (UHI) anomalies with respect to 1990-2014 climatic means during the background period (left panel) and the Chinese New Year (CNY) holiday (right panel). The UHI effects are expressed as (a, b) daily mean surface air temperature (UHI-ΔT_mean_), (c, d) daily maximum surface air temperature (UHI-ΔT_max_), and (e, f) daily minimum surface air temperature (UHI-ΔT_min_) differences between 4 urban stations and 2 non-urban reference stations. The dashed line denotes the linear trend.


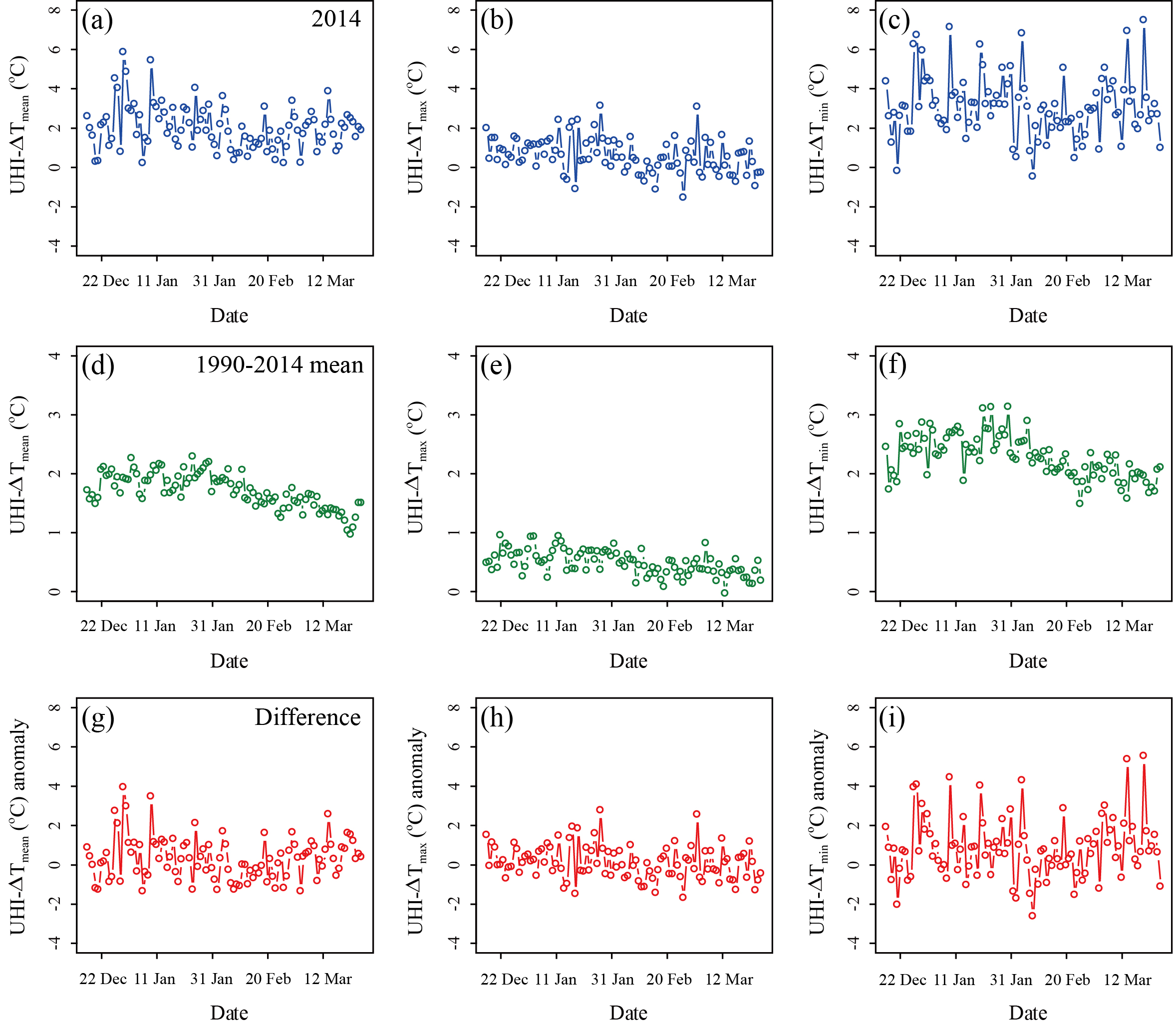


**Figure S3.**  The urban heat island (UHI) values from 17 December to 26 March (day -45 to day +55) in 2014 (upper panel), the UHI values on the same dates as upper panel but averaged over the period of 1990-2014 (middle panel), and their differences (the UHI anomalies in 2014: 2014 minus 1990-2014 mean, lower panel). The UHI effects are expressed as (a, d, g) daily mean surface air temperature (UHI-ΔT_mean_), (b, e, h) daily maximum surface air temperature (UHI-ΔT_max_), and (c, f, i) daily minimum surface air temperature (UHI-ΔT_min_) differences between 4 urban stations and 2 non-urban reference stations.


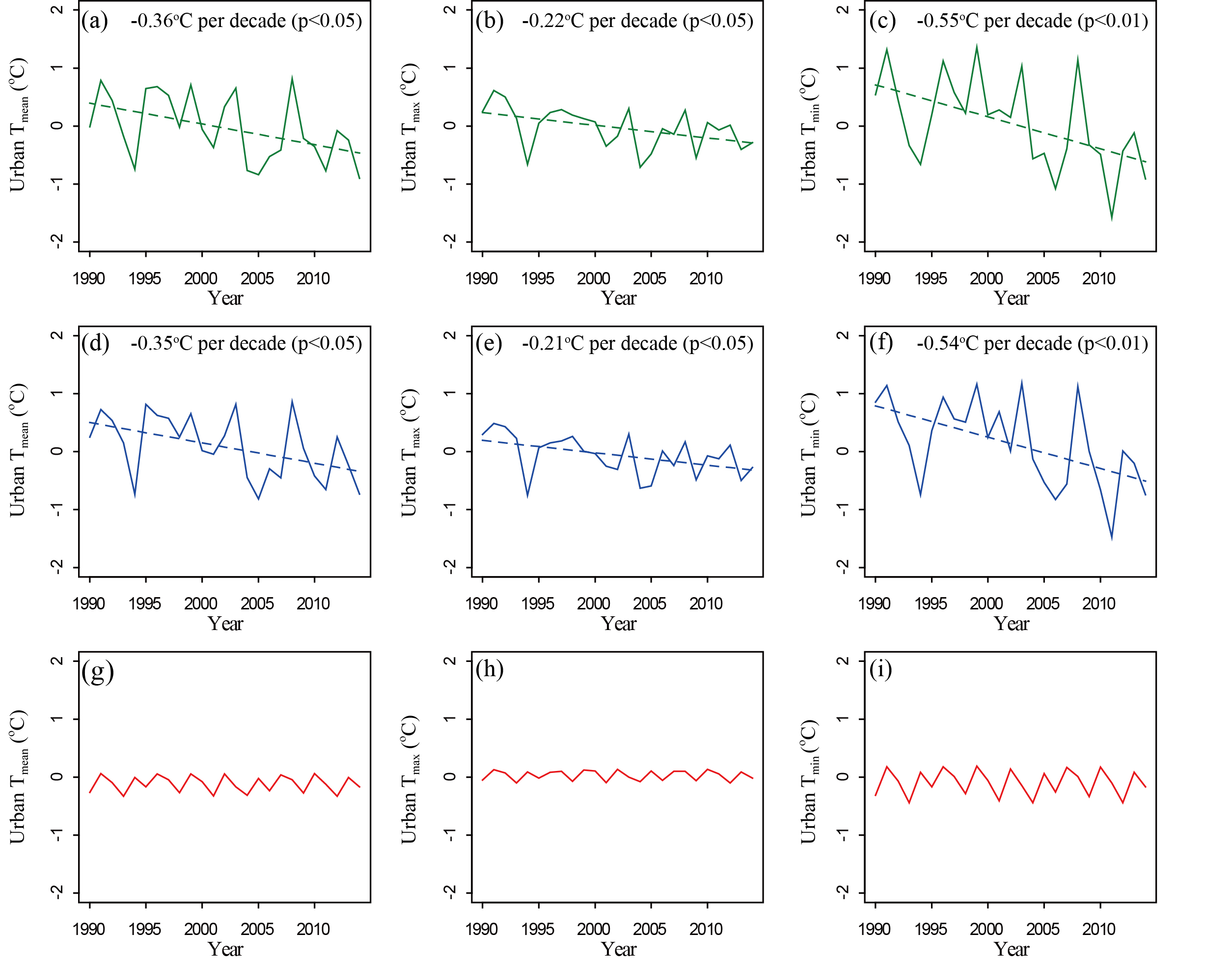


**Figure S4.** Interannual variations of urban daily mean surface air temperature (T_mean_), daily maximum surface air temperature (T_max_), and daily minimum surface air temperature (T_min_) induced by the urban heat island (UHI) differences between the Chinese New Year (CNY) holiday and the background period (CNY holiday minus the background period) with (upper panel) and without (middle panel) the 1990-2014 mean daily UHI values removed, and their differences (removal minus non-removal). The UHI is calculated as surface air temperature difference between 4 urban stations and 2 non-urban reference stations. The urban T_mean_, T_max_, and T_min_ variations are relative to the non-urban values. The dashed line denotes the linear trend. Note that (a)-(c) are the same as (b)-(d) in Fig. 2.
